# Endoscopic aqueductal membrane fenestration was effective for intractable hydrocephalus after removal of a nongerminomatous germ cell tumor exhibiting growing teratoma syndrome: a case report

**DOI:** 10.1186/s12887-022-03743-y

**Published:** 2022-11-28

**Authors:** Sosho Kajiwara, Hideo Nakamura, Kiyohiko Sakata, Satoru Komaki, Tetsuya Negoto, Motohiro Morioka

**Affiliations:** Department of Neurosurgery, Kurume University School of Medicine, 67 Asahimachi, Kurume City, Fukuoka, 830-0011 Japan

**Keywords:** Intracranial growing teratoma syndrome, Nongerminomatous germ cell tumor, Hydrocephalus, Aqueductal membrane, Case report

## Abstract

**Background:**

Primary central nervous system (CNS) germ cell tumors (GCTs) are rare neoplasms predominantly observed in the pediatric and young adult populations. A mixed GCT including immature teratoma exhibiting growing teratoma syndrome is presented. The pathogenesis of growing teratoma syndrome remains unclear, and its treatment strategy has not been established. GCTs are often located within the ventricles, causing hydrocephalus, which sometimes improves after removal of the tumor due to restoration of cerebrospinal fluid (CSF) flow. On the other hand, even if the flow route of CSF from the third ventricle to arachnoid granulations on the brain surface quadrigeminal cistern is restored after removal of the tumor, hydrocephalus may not improve.

**Case presentation:**

A case whose intractable hydrocephalus improved after penetrating the aqueductal membrane via endoscopy is described. An 11-year-old boy was treated for pineal intracranial growing teratoma syndrome (IGTS). The tumor grew rapidly in a short period, and hydrocephalus progressed despite endoscopic third ventriculostomy (ETV). Although the obstruction was removed by radiation, chemotherapy, and total tumor resection, the hydrocephalus did not improve. Endoscopic membrane perforation was performed because a membrane-like structure was seen at the entrance of the cerebral aqueduct on magnetic resonance imaging. The hydrocephalus improved immediately after the operation, and the patient’s consciousness disturbance also improved significantly.

**Conclusion:**

The purpose of this report is to update the current knowledge and standards of management for patients with growing teratoma syndrome, as well as to drive future translational and clinical studies by recognizing the unmet needs concerning hydrocephalus.

## Background

CNS germ cell tumors (GCTs) are rare and account for 3.1% of primary brain tumors in Japan [1]. GCTs are among the most common tumors in children, accounting for about 15% of pediatric brain tumors [2]. The neurohypophysis (hypothalamus/posterior pituitary) and pineal gland are preferred sites of occurrence [3]. Because the treatment strategy differs depending on the pathological diagnosis of GCTs, the optimal treatment strategy should be planned [4]. Intracranial growing teratoma syndrome (IGTS) is a very rare tumor condition, and it is reported to account for 6.5% of all CNS GCTs [5]. IGTS was first reported in 1982 by Logothetis et al. [6], who defined three criteria for IGTS: the tumor markers α-fetoprotein (AFP) and human chorionic gonadotropin (HCG) are normal, but the tumor size continues to increase during or after chemotherapy, and no components of nongerminomatous GCT other than mature teratoma are present. A pineal IGTS that developed with obstructive hydrocephalus, which did not improve even though the obstruction was removed by total tumor resection, is described. However, hydrocephalus improved following penetration of the membranous structure at the entrance of the cerebral aqueduct under endoscopy and subsequent restoration of the normal circulation route for cerebrospinal fluid (CSF). The rare course in this patient and the pathophysiology of hydrocephalus are reported along with a review of the literature.

## Case presentation

**Fig. 1:**
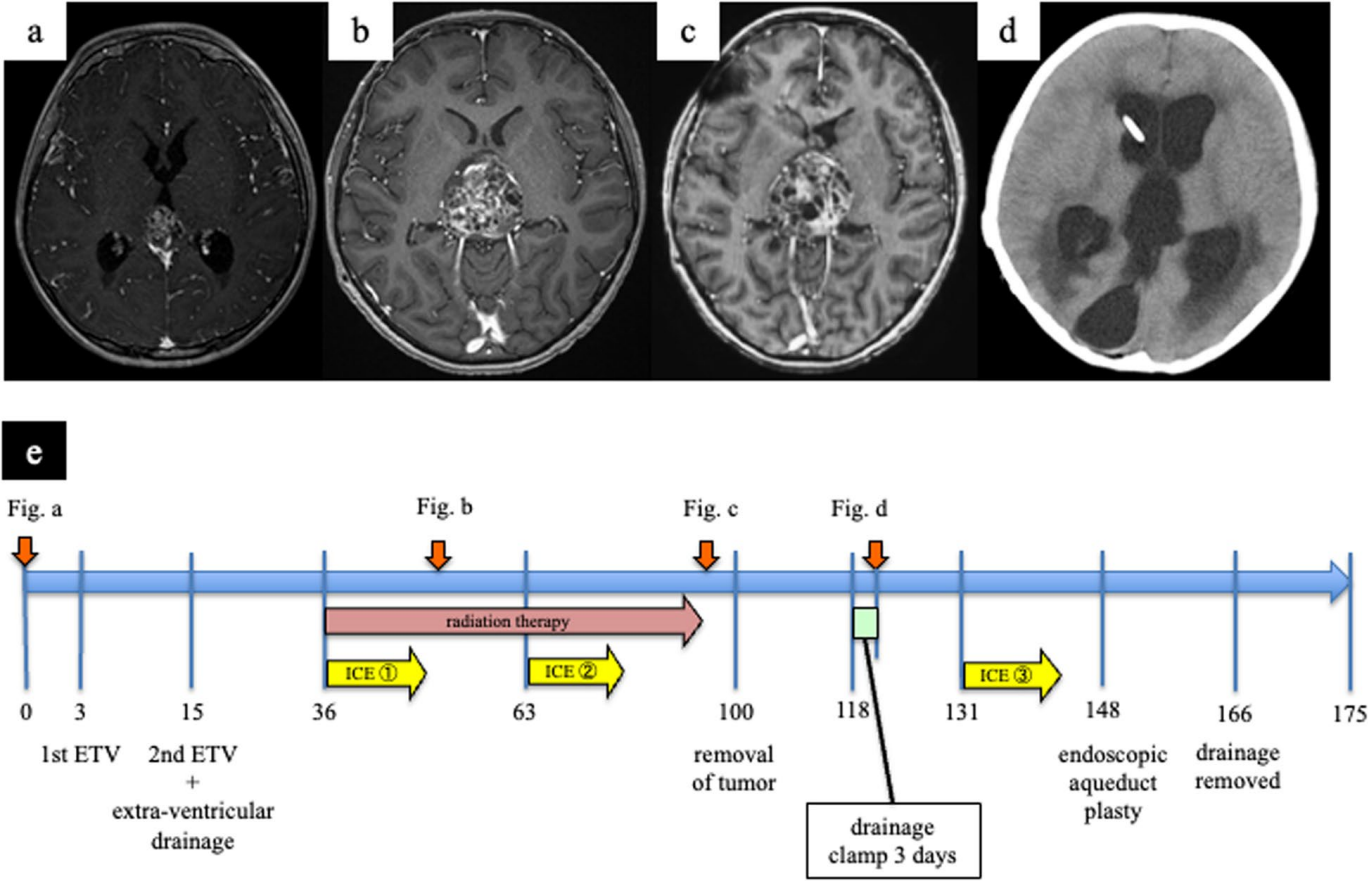
Changes on head T1-enhanced MRI and computed tomography over time. **a** At the time of onset. A heterogeneously enhanced tumor is found in the pineal gland on MRI. **b** After the first cycle of ICE therapy. The tumor has grown and occupies the third ventricle, as seen on MRI. **c** After the second cycle of ICE therapy and radiation therapy. The tumor has grown further, and the pressure on the surrounding brain tissue has increased. In addition, the cystic component is increasing, as seen on MRI. **d** After the clamp test, the ventricles are enlarged. **e** Numbers on the timeline are days after admission

**Fig. 2:**
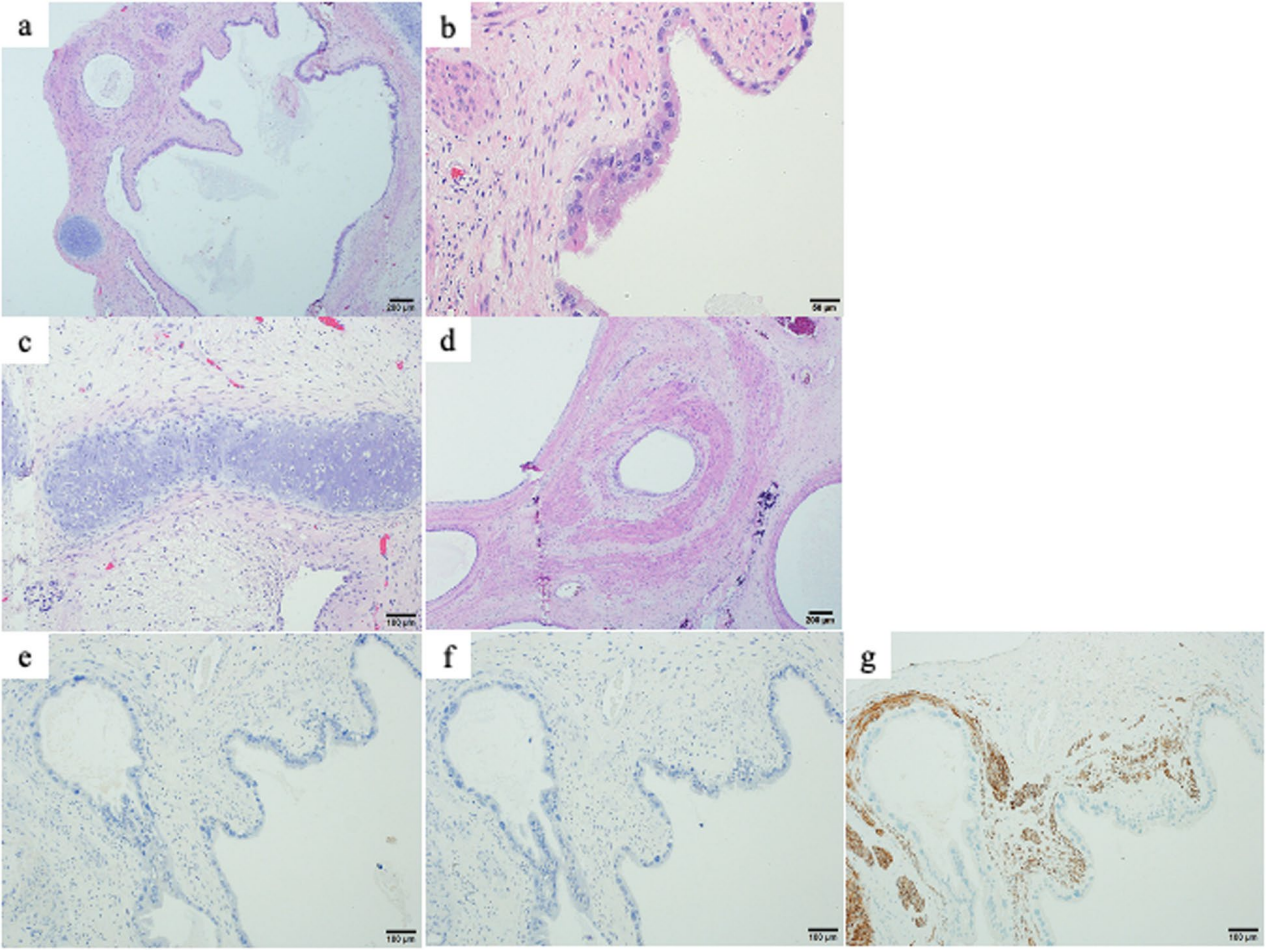
Pathological findings Specimens were examined with an Olympus BX53 polarising light microscope (with UPlanSapo objective lens and WHN10X-H/22 eyepiece lens), digital images were viewed using an Olympus DP73 camera and image analysis software (Olympus cellSens Standard 1.8.1). **a** Overview of hematoxylin and eosin-stained tumor tissue. (× 40). **b** Magnified view shows mucus-secreting columnar epithelium, but no immature components. (× 200) **c**, **d** Hematoxylin and eosin-stained staining of tumor tissue showed cartilage tissue. (**c**, × 100) and calcification and muscle tissue(**d**, × 40). **e**, **f**, **g** No morphologically immature nerve tissue is seen on immunostaining for AFP (**e**, × 100), HCG (**f**, × 100), or PLAP (**g**, × 100).

The timeline from the time of admission is shown in Fig. 1e. An 11-year-old boy with no abnormalities at birth or in development reported visual dysfunction that was accompanied by headache and nausea. Parinaud’s sign and Argyll Robertson pupils were found on neurological examination at admission. T1-weighted gadolinium-enhanced magnetic resonance imaging (MRI) showed a tumor that was heterogeneously enhanced, about 25 mm in size, in the pineal gland occupying the third ventricle (Fig. 1a). Serum AFP was elevated to 104 ng/ml, and serum β-subunit of HCG was within the normal range. Because a consciousness disorder associated with progression of hydrocephalus was observed, endoscopic third ventriculostomy (ETV) was performed with simultaneous biopsy of the tumor 3 days after admission. The pathological diagnosis was a mature teratoma; however, mixed GCT including immature teratoma and yolk sac tumor was suspected because serum AFP was elevated. Because the tumor grew rapidly in a short period, and hydrocephalus progressed despite ETV, ETV was repeated. The fenestration that was opened the first time was unexpectedly obstructed, which instead required the insertion of extraventricular drainage 15 days after admission. Because of the gradual increase in tumor size and the serum AFP value, three cycles of chemotherapy, consisting of ifosfamide, carboplatin, and etoposide (ICE), and radiation therapy (whole ventricle 30.6 Gy + local 19.8 Gy) were initially planned to control the tumor growth. The serum AFP value was within the normal range after the second cycle. However, the tumor size gradually increased to about 50 mm (Fig. 1b, c). Because the patient’s consciousness disturbance was progressing, it was considered that removing the tumor was preferable to continuing the chemotherapy, and therefore, the tumor was removed with an occipital transtentorial approach (OTA) 100 days after admission. Postoperative imaging showed no residual tumor, and a third cycle of ICE therapy was performed. The pathological diagnosis was a mature teratoma, and hematoxylin–eosin staining showed cartilage tissue, calcification, muscle tissue, and epithelial components (Fig. 2a-d). Immunostaining showed no morphologically immature nerves among the tissue that was stained for AFP, HCG, and placental alkaline phosphatase (PLAP) (Fig. 2e-g).

**Fig. 3:**
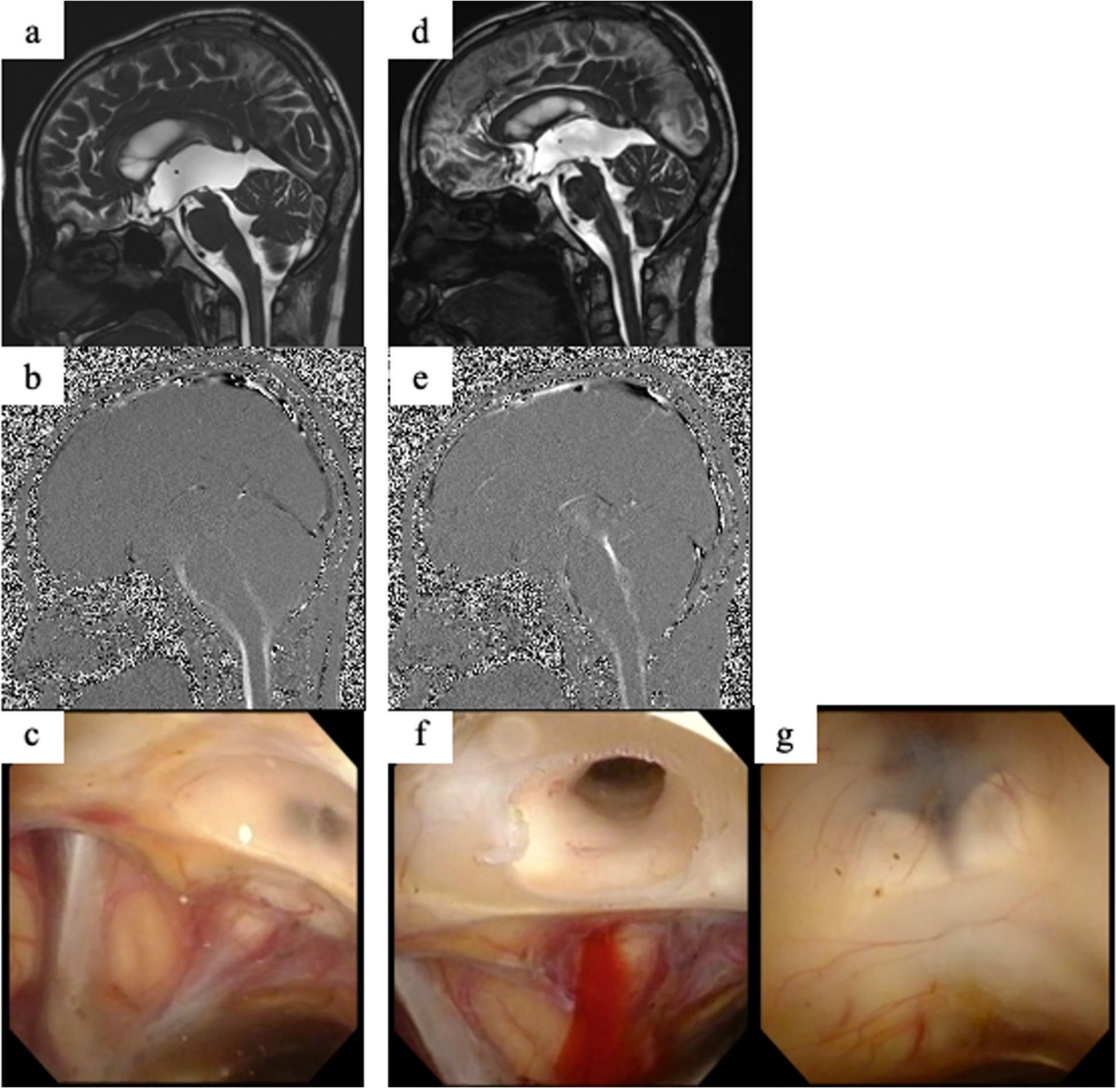
MRI CISS sagittal and endoscopic findings before and after perforating the membrane at the entrance of the cerebral aqueduct. **a**, **b** MRI CISS sagittal view when the extraventricular drain was clamped and consciousness disturbance was prolonged. A septum is found at the entrance of the cerebral aqueduct, and the flow of CSF cannot be confirmed. **c** When observed with an endoscope, a membrane is confirmed at the entrance of the cerebral aqueduct, similar to the MRI findings, and CSF perfusion is not observed. **d**, **e** MRI CISS sagittal view after perforation of the membrane shows that the septum has disappeared, and CSF flow is observed. **f** CSF perfusion is confirmed after perforating the membrane. **g** The bottom of the third ventricle, which was opened by ETV, is closed

Because it was considered that obstruction of CSF flow was restored by tumor removal via the occipital transtentorial route, the clamp test was performed several times to remove the extraventricular CSF drainage tube. However, hydrocephalus did not improve (Fig. 1d), and thus, impaired absorption of CSF was suspected, and it was considered that performing ventricular-peritoneal shunting was necessary. Ventricular cisternography showed that CSF absorption by arachnoid granulation seemed to be not completely impaired, although delayed discharge of contrast was seen, and a membrane-like structure was seen at the entrance of the cerebral aqueduct on MRI constructive interference in steady state (CISS) sagittal imaging (Fig. 3a). It was thought that this membranous structure interfered with the normal flow of CSF (Fig. 3b), and thus, endoscopic membrane perforation was performed 148 days after admission. A white membrane was confirmed at the cerebral aqueduct entrance with a flexible microscope (Fig. 3c). The membrane could be easily perforated with grasping forceps, and when the vacant hole was expanded with a Fogarty balloon catheter, perfusion of CSF was confirmed (Fig. 3f). The hole, which was previously perforated with ETV, was closed again (Fig. 3g); however, no attempt was made to perforate the membrane again because the normal CSF circulation route was already restored. Hydrocephalus improved immediately after the operation, MRI CISS showed that the septum disappeared (Fig. 3d, e), and the patient’s consciousness disturbance also improved considerably. Seventeen days after the endoscopic surgery, the extraventricular drainage could be removed, and he was discharged home.

## Discussion and conclusions

The etiology of IGTS is unknown because its incidence is low. Radiation therapy may play a role, but establishing a relationship between IGTS and radiation therapy is difficult [7, 8]. Chemotherapy and radiotherapy may remove the immature component, but the mature component may remain; chemotherapy may also induce transformation of the immature component to the mature component [9]. Histological diagnosis is important for deciding the treatment strategy, but early removal may improve the outcome or evoke dissemination when the tumor tends to grow in patients with mixed GCT. Thus, determining the optimal timing of excision is very important. In this case, it appears that it was possible to remove the tumor at the right time. There was the option of removing the tumor when the tumor tended to grow, but dissemination may be prevented at the time of removal when the patient first undergoes radio-chemotherapy [2]. Since the initial biopsy results showed the presence of an immature component, it is quite possible that radio-chemotherapy prior to removal would have normalized serum tumor markers and reduced the risk of dissemination of the immature components by surgery. IGTS should be considered if a mismatch is seen between serum marker levels and tumor size on MRI [10]. Frequent imaging follow-up may be necessary. In addition, complete removal is necessary to cure IGTS, and the best timing for removal may be before it grows and symptoms occur, if possible [5, 11, 12].

There are several typical surgical approaches to pineal tumors, including OTA, the supra-cerebellar infra-tentorial approach, and the transventricular approach using an endoscope. In the present case, OTA was the most suitable approach because the tumor had grown rapidly, and a wide view was required to completely remove the large tumor.

The most important point is that a good outcome can be obtained without performing ventriculoperitoneal shunting by restoration of normal CSF perfusion to treat the hydrocephalus that persisted after IGTS removal.

The membrane that formed at the cerebral aqueduct entrance after tumor removal may have been formed by physical obstruction due to tumor growth, stagnation of CSF flow, inflammation due to surgery, and fibrosis associated with radio-chemotherapy [13]. According to previous reports, a membrane is formed by increased numbers of fibroblasts due to inflammation and trauma, in addition to congenital factors [14], which may include transforming growth factor-β [15]. Another report described stoma closure due to tumor dissemination [16], but no obvious dissemination findings were seen. Although the possibility that the membrane may have formed due to long-term extraventricular drainage cannot be excluded, the membrane was thought to have formed due to a combination of factors including tumor growth, radiation chemotherapy, and surgery. Because the choroid plexus of the fourth ventricle produces CSF, which drains via the cerebral aqueduct to extra-ventricular drainage, the CSF near the membrane formation was unlikely to be stagnant. It is also necessary to consider the possibility of hematoma components being involved in the mechanism of occlusion. After tumor removal, the region from the extraction cavity to the entrance of the cerebral aqueduct was washed thoroughly, and the hematoma components were also excluded thoroughly. In addition, postoperative MRI T2*-weighted imaging did not show any low-intensity areas around the entrance of the cerebral aqueduct that were suspicious of hemosiderosis. Moreover, there was no xanthochromia suggesting blood contamination in the CSF examination during extra-ventricular CSF drainage or endoscopic surgery. Therefore, there appeared to have been no hematoma involvement in the membrane formation at the entrance of the cerebral aqueduct.

We hypothesize that hydrocephalus after tumor surgery is often caused by impaired absorption of arachnoid granulations, but evaluation of the cause of hydrocephalus is important. Imaging modalities such as MRI CISS are sometimes useful to identify the obstruction point, and this type of imaging is also effective for pre- and postoperative evaluation because it can evaluate the flow of CSF [17]. Physicians should investigate what interferes with normal CSF circulation in tumor-related hydrocephalus, rather than immediately perform ETV or shunting.

Previously, CSF was thought to be produced in the choroid plexus, circulate unidirectionally through the arachnoid granulation, and be absorbed. However, CSF is currently thought to move bidirectionally between the CSF cavity and the interstitial fluid of the surrounding brain tissue. [18]. Moisture, which accounts for 99% of CSF, is believed to move bidirectionally via aquaporin-4 in the ependymal cells around the cerebrospinal capillaries and periventricular regions, and only 1% diffuses in all directions of the CSF cavity. In the present case, the tumor was removed with an occipital transtentorial approach, and if the new CSF theory is correct, the risk of hydrocephalus seems to be low because the CSF flows extensively from the third ventricle to the brain surface. However, the absorption capacity was insufficient, and consideration of the old CSF theory, including flow through the cerebral aqueduct, was important.

Schroeder et al. reported that endoscopic aqueduct plasty is effective for membranous occlusion of the cerebral aqueduct [19], and it was also effective in the present case. When endoscopic aqueduct plasty is performed, ETV is often added in case of re-occlusion, but it was judged that ETV was not necessary because no stenosis of the cerebral aqueduct was present, and sufficient space was secured at the cerebral aqueduct entrance.

Although the effectiveness of shunting has been established, Mallucci et al. reported that about 20% of all types of shunts require shunt revision, a rate that is not low [20]. It is important that a good outcome can be obtained in children without shunting, and shunt-free treatments should be sought.

Although a few case reports of IGTS have been published, this is the first report to describe hydrocephalus after performing effective surgery for IGTS. It was suspected on MRI that a membrane had formed, and that spinal fluid perfusion was impaired. However, it was doubtful that perforating the membrane was enough to improve the hydrocephalus, and it was discussed among the medical team many times. Finally, a challenging approach was attempted to obtain a good outcome without shunting, considering that the patient was a child and his future growth and life. It was very novel that it was possible to achieve this, and it is necessary to accumulate more cases and reconsider the necessity of shunt insertion in the future.

In closing, a case of IGTS for which determination of the optimal timing of excision was difficult was presented. Although IGTS basically requires excision, neoadjuvant therapy before excision may eliminate malignant components and prevent dissemination at the time of excision. Thus, the timing of removal should be carefully considered in each case. Establishing normal CSF circulation is important when hydrocephalus occurs, because creating one route from the third ventricle to the posterior part of the brain surface was not effective in the present case.

## Data Availability

All data generated or analyzed during this study are included in this article. Further enquiries can be directed to the corresponding author.

## Abbreviations

AFP: α-Fetoprotein
CISS: Constructive interference in steady state
CNS: Central nervous system
CSF: Cerebrospinal fluid
ETV: Endoscopic third ventriculostomy
GCTs: Germ cell tumors
HCG: Human chorionic gonadotropin
IGTS: Intracranial growing teratoma syndrome
MRI: Magnetic resonance imaging
OTA: Occipital transtentorial approach
PLAP: Placetal alkaline phosphatase
